# Triage vital signs predict in-hospital mortality among emergency department patients with acute poisoning: a case control study

**DOI:** 10.1186/1472-6963-12-262

**Published:** 2012-08-18

**Authors:** Jiun-Hao Yu, Yi-Ming Weng, Kuan-Fu Chen, Shou-Yen Chen, Chih-Chuan Lin

**Affiliations:** 1 Department of Emergency Medicine, Chang Gung Memorial Hospital and Chang Gung University College of Medicine, Linkou, No. 5, Fu-Hsing St., Kuei Shan Hsiang, Tao-yuan Hsien, Taiwan; 2 Department of Emergency Medicine, Chang Gung Memorial Hospital at Keelung and Chang Gung University, Taoyuan, Taiwan

## Abstract

**Background:**

To document the relationship between triage vital signs and in-hospital mortality among emergency department (ED) patients with acute poisoning.

**Methods:**

Poisoning patients who admitted to our emergency department during the study period were enrolled. Patient’s demographic data were collected and odds ratios (OR) of triage vital signs to in-hospital mortality were assessed. Receiver operating characteristic curve was used to determine the proper cut-off value of vital signs that predict in-hospital mortality. Logistic regression analysis was performed to test the association of in-hospital mortality and vital signs after adjusting for different variables.

**Results:**

997 acute poisoning patients were enrolled, with 70 fatal cases (6.7%). A J-shaped relationship was found between triage vital signs and in-hospital mortality. ED triage vital signs exceed cut-off values independently predict in-hospital mortality after adjusting for variables were as follow: body temperature <36 or >37°C, *p* < 0.01, OR = 2.8; systolic blood pressure <100 or >150 mmHg, *p* < 0.01, OR: 2.5; heart rate <35 or >120 bpm, *p* < 0.01, OR: 3.1; respiratory rate <16 or >20 per minute, *p* = 0.38, OR: 1.4.

**Conclusions:**

Triage vital signs could predict in-hospital mortality among ED patients with acute poisoning. A J-curve relationship was found between triage vital signs and in-hospital mortality. ED physicians should take note of the extreme initial vital signs in these patients.

## Background

In the modern practice of medical toxicology, vital signs play an important role in diagnosis since they are the key components of toxic syndromes. However, their role in assessing severity of poisoned patients is still lack of evidence. Most of the previous research focused on the relationship between a single specific poison and its prognostic factors, such as tachycardia in glyphosate-surfactant intoxication or low body temperature in paraquat intoxication. These reports are of little use when you face patients with mixed drug poisoning or unknown poison [1,2]. Several scoring systems were also developed to predict in-hospital mortality for certain herbicides. Glasgow Coma Scale (GCS), Acute Physiology and Chronic Health Evaluation (APACHE) II scores, and Simplified Acute Physiology Score (SAPS) II are some of the examples [3-6]. These three scoring systems were compared each other and found that they had similar associations with mortality [4]. The modified APACHE II system may be of value to predict mortality in organophosphate poisoning patients in an emergency situation [3]. A SAPS II score above 11 within the first 24 hours is a predictor of poor outcome in patients with acute organophosphate poisoning [5]. However, these scoring systems were developed in an intensive care unit for herbicide intoxication and might not be suitable for use with general acute poisoning patients in an emergency department (ED) setting. Patient with acute poisoning in ED may have mixed drug intoxication or have changes in consciousness and be unwilling or unable to give an exact history. Besides, it may be difficult to make further decisions regarding these poisoned patients, such as whether to admit them to the intensive care unit or the general ward versus safely discharging them. Knowing the relationship between initial vital signs at ED triage and in-hospital mortality of acute poisoning patients may alerts ED physicians and can assist them in decision making. Therefore, it is our purpose to assess if triage vital signs could be a useful tool to assess severity of poisoning patients in the emergency department.

In another aspect, the prognosis of acute poisoning depends on the exposure of toxin, the amount of toxin ingestion and the physiology of compensation. We conducted this retrospective case–control study to test the hypothesis that the initial vital signs at ED triage, which stood for physiology response after acute poisoning, could serve as a reliable indicator of in-hospital mortality.

## Methods

### Study design and settings

This was a study conducted at a university-affiliated teaching hospital with an estimated annual ED volume of 227,000 visits. All the patients whose initial impression were acute poisoning or patients who were found out to be poisoned patients were registered in a database. Consecutive poisoned patients in the database who presented between January 1, 2005, and December 31, 2008 were then enrolled into this study. This study was approved by the Institutional Review Board (the”IRB”) of Chang Gung Medical Foundation on 2008/12/31. The IRB is organized and operates according to Good Clinical Practice and the applicable laws and regulations.

### Patient population

All the enrolled patients were at least 18 years of age. Patients who suffered from acute poisoning via ingestion or inhalation were recorded by front-line physician using electronic medical system.

### Study protocol

Trained study assistants who were blinded to the study purpose performed the chart review and data abstraction using standardized template with clear definition and code. Data was retrieved via ED electronic medical records. The first authors performed a quality improvement feedback after the data analysis during the study by holding periodic meetings with assistants.

A physician reviewed the electronic medical records of patients who met the inclusion criteria during the study period and examined the data. Patients with incomplete records, wrong implementation of the code, or traumatic patients were then excluded. A reviewer analyzed the ingested toxins and confirmed the ingestions. Patients were divided into the surviving and the fatal group. For all eligible patients, demographic data were collected including age, gender, toxic agents, psychiatric medical history, suicide attempt, and days of hospital stay. The vital signs were recorded at triage area when patients on arrival to the ED. The vital signs recorded included systolic blood pressure (SBP), diastolic blood pressure (DBP), heart rate (HR), respiratory rate (RR), and body temperature (BT). The mixed poison agents defined as more than two categories of poison ingestion. Decontamination, antidote, and life support management were applied by discretion of emergency physicians. Poisoning patients who attempted suicide also received psychiatric consultation by the law to assess their psychiatric medical histories for preventing recurrent suicide.

To assess the association of in-hospital mortality and ED vital signs, univariate analysis and logistic regression were them performed after the above variable collected.

### Outcome measurements

The primary outcome was in-hospital mortality, including patients die in ED, ward, or intensive care unit. The relationships between initial vital signs and in-hospital mortality were shown by odds ratio of different strata of vital signs, including 10 mmHg strata for systolic blood pressure, 1°C strata for body temperature, 10 beats per minute strata for heart rate, and 4 per minute strata for respiratory rate.

### Statistical analysis

Data were analyzed using SPSS 13.0 for Windows (SPSS, Chicago, IL). Demographic and clinical characteristics of patients were summarized by descriptive statistics. Continuous data are presented as means ± standard deviation (SD) or median with interquartile range (IQR) when appropriate. Categorical data are reported as number and percentage (%). The comparison between the surviving and fatal group was analyzed with a *t*-test or Wilcoxon rank-sum test for continuous variables and the Pearson chi-square test or Fisher’s exact test for categorical variables when appropriate. In all analyses, *P* <  0.05 indicated statistical significance. Variables those with *p*-value less than 0.02 in the univariate analyses will be considered as potential predictors.

A receiver operating characteristic (ROC) curve was used to determine the proper cut-off value of vital signs that predicted in-hospital mortality. The best cut-off point was that which maximized the sum of specificity and sensitivity in the ROC analysis. The multivariate logistic regression analysis will be utilized to evaluation the potential confounding, effect modification or mediation between potential predictors and the mortality

## Results

**Figure 1:**
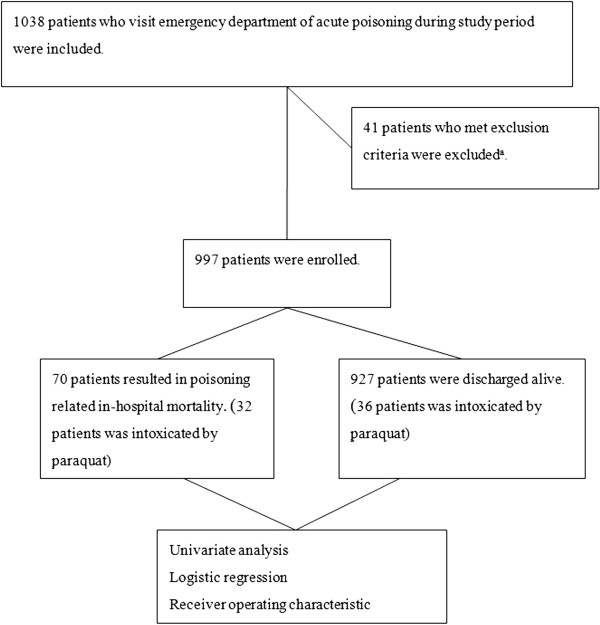
**Study protocol and patient grouping.**^a^ Patients were excluded due to missing data, wrong registration or traumatic patients.

A total of 1038 patients were eligible during the four-year study period, but 41 patients were excluded due to missing data or wrong registration. Subsequently, 997 acute poisoning patients were enrolled (Figure 1).

### Differences in demographic characteristics and poison agents between groups

Table 1 summarizes the demographic characteristics and the results of univariate analysis for fatalities and survivors among the enrolled patients. The 70 fatal cases (6.7%) showed male predominance (72.9 vs. 48.8%, *p* < 0.01), lower body temperature (36.1 ± 1.2 vs. 36.4 ± 0.8°C, *p* = 0.03), and tachycardia (101.6 ± 29.3 vs. 92.2 ± 23.4 bpm, *p* = 0.01) compared with the surviving group. No significant difference in the mean age, triage respiratory rate, triage blood pressure, suicide attempts, psychiatric medical histories, and length of hospital stay were found between the groups. We identified the patient with mixed agent poisoning and took them into statistical analysis and be one of the variable. The fatal group has less mixed agent poisoning (n = 3, 4.2%) compared with the surviving group (n = 106, 11.4%) but there is no significant difference (p = 0.073). The three fatal cases were intoxicated by paraquat and amphetamine, organophosphate and benzodiazepine, and organophosphate and caustic agents.

**Table 1:** Demographics characteristics between fatal and surviving groups

	**Fatalities (N = 70)**	**Survivors (N = 927)**	***p*- value**
**Mean age in years (SD)**	50.9 (17.8)	46.4(19.4)	0.06*
**Male Gender, n (%)**	51(72.9)	451(48.8)	<0.01*
**Mean BT, °C (SD)**	36.1(1.2)	36.4(0.8)	0.03*
**Mean HR, beats per minute (SD)**	101.6(29.3)	92.2(23.4)	0.01*
**Mena RR, per minute (SD)**	19.9(4.2)	19.1(2.9)	0.10*
**Mean SBP, mmHg (SD)**	135.8(40.9)	134.8(28.3)	0.85
**Mean DBP, mmHg (SD)**	74.5(23.7)	77.9(16.8)	0.24*
**Mean LOS, days (SD)**	10.5(12.6)	9.0(11.5)	0.31*
**Suicide attempt, n (%)**	49(70.0)	584(63.0)	0.24
**Psychiatric medical history, n (%)**	15(21.4)	294(31.7)	0.07
**Mixed poison agents, n (%)**	3(4.2)	106(11.4)	0.07

Abbreviations: SD, standard deviation; BT, body temperature; HR, heart rate; RR, respiratory rate; SBP, systolic blood pressure; DBP, diastolic blood pressure; MAP, mean arterial pressure; LOS, length of stay in hospital.

Continuous variables (age, BT, HR, RR, SBP, DBP, LOS) were represented as mean ± standard deviation (SD), and were tested using independent t-test.

Number (n) and percentage (%) represented categorical data (male gender, suicide attempt, psychiatric medical history), and Chi-square test was used as indicated.

Case fatality means in-hospital mortality.

*The p value is < 0.05 after excluding the patients with paraquat intoxication.

Different poison agents among the fatal and surviving groups are shown in Table 2. In order to present the character of individual poison agents, we excluded the mixed poison agents, including three patients in fatal group and 32 patients in surviving group. The most common lethal agents were paraquat (N = 31, 46.3%), caustic agents (N = 7, 10.4%), digoxin (N = 6, 9.0%), and organophosphate (N = 4, 5.9%). The lethal agent associated with high odds ratio of in-hospital mortality was paraquat (OR 22.5, 95% CI 12.4-40.7), followed by carbamate (OR 13.7, 95% CI 1.9-99.1), amphetamine (OR 6.9, 95% CI 1.2-38.1), and digoxin (OR 4.8, 95% CI 1.8-12.5). Significant difference in the paraquat, carbamate, digoxin, and hypnotics were found between the fatal and surviving groups.

**Table 2:** Different poison agents among fatal and surviving groups

	**OR (95% CI)**	**Fatalities (N = 67,%)**	**Survivors (N = 895,%)**	***p*- value**
**Paraquat**	22.5 (12.4-40.7)	31 (46.3)	33 (3.7)	<0.01
**Carbamate**	13.7(1.9-99.1)	2 (2.9)	2 (0.2)	0.03
**Amphetamine**	6.9 (1.2-38.1)	2 (2.9)	4 (0.4)	0.06
**Digoxin**	4.8 (1.8-12.5)	6 (9.0)	18 (2.0)	<0.01
**Methanol**	3.4 (0.37-30.6)	1 (1.5)	4 (0.4)	0.30
**Opioid**	3.0 (0.8-10.5)	3 (4.5)	14 (1.6)	0.11
**Organophosphate**	1.4 (0.48-4.02)	4 (5.9)	39 (4.3)	0.53
**Glyphosate**	1.8 (0.4-8.1)	2 (2.9)	15 (1.7)	0.33
**Alcohol**	1.7 (0.4-7.5)	2 (2.9)	16 (1.8)	0.36
**Caustic agents**	1.1 (0.5-2.6)	7 (10.4)	83 (9.3)	0.68
**CO**	0.3 (0.04-1.87)	1 (1.5)	50 (5.6)	0.15
**Warfarin**	0.3 (0.04-2.1)	1 (1.5)	45 (5.0)	0.37
**Hypnotics**	0.07 (0.01-0.48)	1 (1.5)	168 (18.8)	<0.01

Abbreviations: OR, odds ration; CO, carbon-monoxide.

All variables were presented with 95% confidence interval (95%CI), number (n), percentage (%), and Chi-square test was used.

The poison agents in the table are in order of odds ratio. Among them, paraquat has the highest OR.

Case fatality means in-hospital mortality.

# In order to present the character of individual poison agents, we excluded the mixed poison agents. Three patients in fatal group and 32 patients in surviving group were excluded in this table.

### The association between ED triage vital signs and poison-related in-hospital mortality

**Figure 2:**
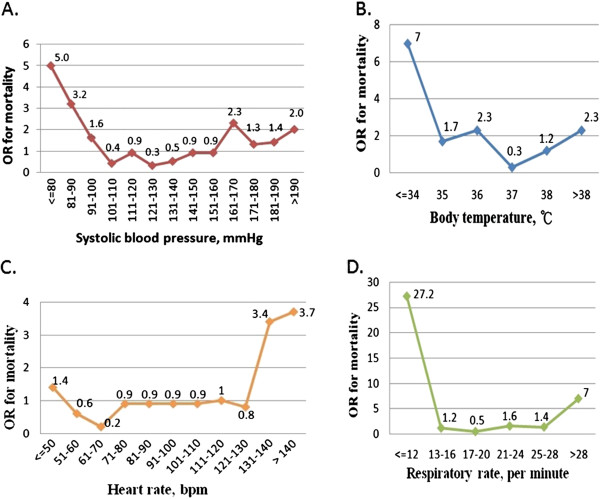
The odds ratio (OR) in different strata of initial vital signs at emergency department triage, including (A) systolic blood pressure, (B) body temperature, (C) heart rate, and (D) respiratory rate.

The odds ratios (OR) of in-hospital mortality for SBP, BT, HR, and RR revealed J-shaped relationships (Figure 2). Patients with an SBP of more than 190 mmHg or less than 100 mmHg had a greater than two-fold increase in the OR for in-hospital mortality, respectively. Initial BT of less than 34°C or over 38°C showed seven- and two-fold increased OR for in-hospital mortality, respectively. A triage HR of below 50 bpm or above 120 bpm was associated with increase in OR for in-hospital mortality, respectively. RR >28 or <12 per minute was associated with higher odds of in-hospital mortality (RR <12, OR = 27.2; RR >28, OR = 7). The patients with extremely abnormal vital signs had the greatest risk of in-hospital mortality. Therefore, further analysis was performed to find out the proper cut-off values to predict the in-hospital mortality.

**Figure 3:**
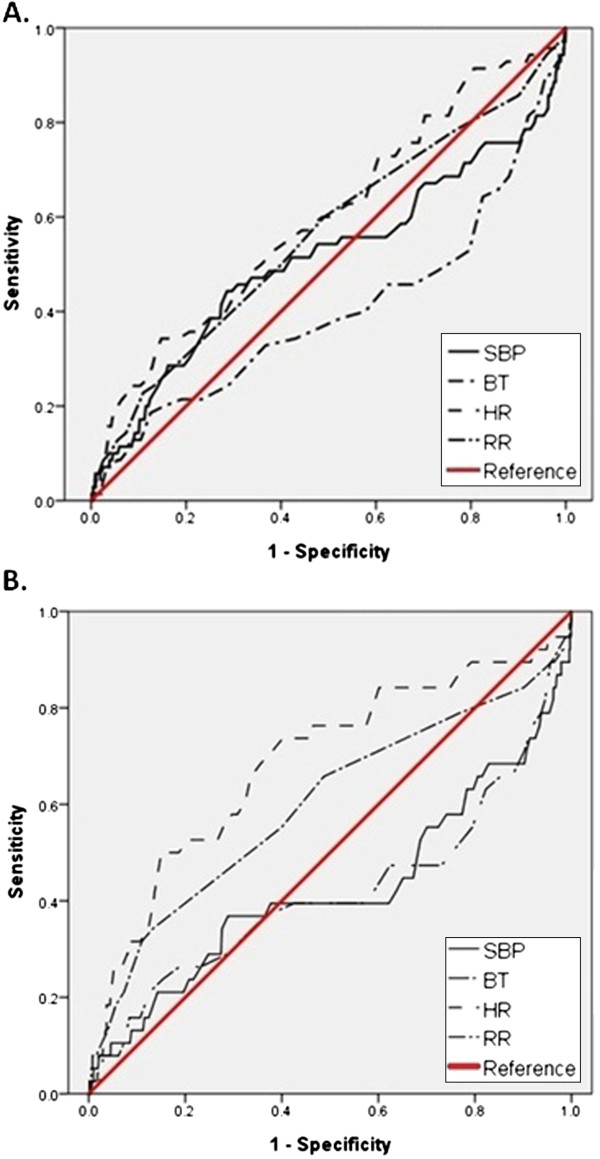
**Receiver operating characteristic (ROC) curve for systolic blood pressure (SBP), body temperature (BT), heart rate (HR), and respiratory rate (RR).** # Patients with paraquat intoxication were excluded in the figure B.

By constructing a receiver operating characteristic (ROC) curve, we plotted the true-positive rate (sensitivity) against the false-positive rate (1-specificity) at each point (Figure 3). The optimum cut-off points using triage vital signs to predict in-hospital mortality were BT <36 or >37°C, SBP <100 or >150 mmHg, HR <35 or >120 bpm, RR <16 or >20 per minute (Figure 3A).

After the univariate analysis, logistic regression analysis was performed (Table 3). ED triage vital signs exceeding cut-off values independently predicted in-hospital mortality after adjusting for variables (BT <36 or >37°C, OR 2.8, 95%CI 1.5 – 5.3, *p* < 0.01; SBP <100 or >150 mmHg, OR 2.5, 95%CI 1.4 – 4.7, *p* < 0.01; HR <35 or >120 bpm, OR 3.1, 95%CI 1.5 – 6.6, *p* < 0.01; RR <16 or >20 per minute, OR 1.4, 95%CI 0.7 – 2.9, *p* = 0.38).

**Table 3:** Logistic regression analysis of predictors associated with poison-related mortality

	**OR (95%CI)**	***p*-value**
**Male gender**	1.9 (0.9– 3.7)	0.07
**Mean BT < 36 or >37, °C**	2.8 (1.5 – 5.3)	<0.01
**Mean SBP < 100 or >150, mmHg**	2.5 (1.4– 4.7)	<0.01
**Mean HR < 35 or >120,bpm**	3.1 (1.5– 6.6)	<0.01
**Mean RR < 16 or >20, per minute**	1.4 (0.7– 2.9)	0.38
**Paraquat**	28.5 (13.8– 58.8)	<0.01
**Carbamate**	7.7 (0.8– 72.3)	0.08
**Digoxin**	13.2 (4.4– 40.2)	<0.01
**Hypnotics**	0.2 (0.03 – 1.69)	0.15

Abbreviations: OR, odds ration; SBP, systolic blood pressure; HR, heart rate; RR, respiratory rate; BT, body temperature.

# After excluding the paraquat poisoning patients, the ED triage vital signs exceeding cut-off values independently predicted in-hospital mortality after adjusting for variables were as follow:BT <36 or >37°C, OR 3.2, 95%CI 1.4 – 7.1, *p* < 0.01; SBP <100 or >150 mmHg, OR 2.2, 95%CI 1.0 – 4.5, *p* = 0.04; HR <35 or >120 bpm, OR 2.7, 95%CI 1.2 – 6.0, *p* = 0.01; RR <16 or >20 per minute, OR 2.4, 95%CI 1.0 – 5.1, *p* = 0.03.

### The impact of paraquat in the study

Among the 70 fatal cases, 32 patients (45.7%) were intoxicated by paraquat. Nearly half (n = 32/68, 47%) of paraquat poisoning cases was fatal and has high odds ratio of mortality. Identifying the paraquat itself had a higher predictive value than the vital signs. To diminish or realize the impact of paraqaut in this study, we excluded the paraquat poisoned patients and re-conducted the statistic analysis. In Table 1, the mean age (p = 0.01), male gender (p < 0.01), mean body temperature (p < 0.04), mean heart rate (P < 0.01), mean respiratory rate (p < 0.01), mean diastolic blood pressure (p < 0.01), and length of hospital stay (p < 0.01) between the fatal and surviving groups revealed statistically significance after excluding the patients with paraquat intoxication. We constructed the ROC curve (Figure 3B) and found that the cut-off values to predict in-hospital mortality are nearly the same with total poisoning population. Logistic regression analysis was performed and the ED triage vital signs exceeding cut-off values independently predicted in-hospital mortality after adjusting for variables (BT <36 or >37°C, OR 3.2, 95%CI 1.4 – 7.1, *p* < 0.01; SBP <100 or >150 mmHg, OR 2.2, 95%CI 1.0 – 4.5, *p* = 0.04; HR <35 or >120 bpm, OR 2.7, 95%CI 1.2 – 6.0, *p* = 0.01; RR <16 or >20 per minute, OR 2.4, 95%CI 1.0 – 5.1, *p* = 0.03).

## Discussion

Our results suggest the presence of a J-curve relationship between triage vital signs and in-hospital mortality among acute poisoning patients in the emergency department. We also established optimum cut-off points of triage vital signs to predict in-hospital mortality. This concept is modest and particularly helpful for front-line emergency physicians. ED physicians should be aware of vital signs that exceed the cut-off values in acute poisoning patients.

Prognostic factors that help to predict overall poisoning-related fatality have rarely been elucidated. Suicidal intent, ingestion of paraquat, abnormal vital signs , mixed drug intoxication, and old age have been found to be useful predictors in predict poisoning patients’ mortality [7,8]. Hu et al. found that factors such as herbicide poisoning, hypotension, and respiratory failure upon presentation can predict overall poisoning-related fatality in ED settings [9]. Jayashree et al [10]. reported hypotension at admission as the most significant predictor of death in children admitted to the ICU with acute poisoning. To our knowledge, no prior study has examined the relationship between triage vital signs and in-hospital mortality in overall acute poisoning patients. Although it is premature to conclude based on the present evidence that the cut-off values of the observed J-shaped curve would be the same in different ED settings, it appears reasonable to apply risk stratification in acute poisoning patients.

Half of the deaths in this study were due to paraquat poisoning. To realize the impact of paraquat in this study, we excluded the paraquat poisoning patients and repeated the analysis. Although excluding the paraquat may change the variation of triage vital signs, it still can predict in-hospital mortality in the further analysis. In addition to well-recognized lethal agents, such as paraquat poisoning, our study identified the ingestion of carbamate (OR: 13.7), amphetamine (OR: 6.9), or digoxin (OR: 4.8) as significantly associated with poisoning-related fatality. Patients taking paraquat, carbamate, amphetamine, or digoxin may predict mortality better than their abnormal vital signs.

The most common lethal agents in study were paraquat, organophosphate, and digoxin. As we known, these agents may result in bradycardia more than tachycardia. However, tachycardia is more prominent in fatal group compare to survived cases in our study (Table 1). The reason are as follow:In paraquat intoxicated patients, direct cardiovascular toxicity, hypoxia, hypotension or increased sympathetic tone may contribute to tachycardia [11,12]. In organophosphate poisoning patients, there are three phases of cardiotoxicity according to Ludomirsky et al [13]. : (1) a brief period of intense increased in sympathetic tone manifested by sinus tachycardia; (2) a prolonged phase characterized by parasympathetic “outflow” and manifested by AV conduction disturbances; and (3) a phase in which QT-interval prolongation, pleomorphic tachycardia, and sudden cardiac death are characteristic. The third phase is fetal and can appear unexpectedly after exposure. With toxic concentrations of digitalis, stimulation of sympathetic nerve activity may also occur and is dangerous. The manifestations included complex supraventricular dysrhythmias, bidirectional ventricular tachycardia, and ventricular tachycardia [14]. Therefore, tachycardia may be prominent when patients presented to ED with paraquat, organophosphate, or digoxin intoxication. Besides, mixed agent poisoning accounted for ten percent of population in this study. There were multiple different toxic agents with a wide range of presentation. Patients who take tricyclic antidepressants and propranolol may present with hypotension without tachycardia or bradycardia. In this situation, the severity model in this study may help to find out the patient with great risk of in-hospital mortality easily and quickly.

The mortality rate of poisoning varies significantly in different countries and is influenced by many factors. Mortality rates of poisoning in the general population have been reported as 0.24% in Germany [6], 1.4% in Hong Kong [15], 2.9% in Greece [16], 5.7% in Taiwan [17], and 8% in Sri Lanka [18]. In an aging population, mortality is much higher than that in the younger general population. One study reported that poisoned patients >65 years old had a mortality rate of 9.6% in Taiwan [7]. In our study, the mortality rate in patients >18 years old was 6.7%. The high mortality rate in this study might be due to the wide use of highly toxic agrochemicals in Taiwan. Also, our study was held in a tertiary medical center, which may result in overestimation of the mortality rate of the general poisoning population.

In this study, we did not focus on ingestion of a single poison agent because patients could present to the ED with mixed drug ingestion, multi-toxin exposure, inexact present histories, or different exposure time. Instead, we attempted to identify acute poisoning patients with greatest risk of in-hospital mortality at the triage. Although the different characters of poison agents may change the variation of “Triage vital signs”, it remained significant associated with in-hospital mortality after adjusting for specific agents by logistic regression.

Several limitations of this study warrant discussion. First, our study was retrospective, and the data were collected from a computerized database and chart review. Although we made every effort to remain objective, possible errors may have occurred. Second, this study was conducted in a university-affiliated teaching hospital, which may limit the generalizability of our findings. A comparative study with other systems would be of interest. Third, most toxic agents were categorized according to the clinical signs and symptoms and history of toxin exposure without any qualitative or quantitative laboratory tests. However, this method was adopted from previous studies. Fourth, multicenter study should be done to evaluate its efficacy for predict mortality rate.

## Conclusions

Triage vital signs could predict in-hospital mortality among ED patients presenting with acute poisoning. A J**-**curve relationship was found between triage vital signs and in-hospital mortality. ED physicians should take note of the extreme initial vital signs in these patients.

## Competing interests

No any financial and personal relationships with other people or organizations that could inappropriately influence (bias) the work.

## Authors’ contributions

Jiun-Hao Yu participated in the analysis of data and drafted the manuscript. Chih-Chuan Lin participated in the design of the study and gave final approval of the version to be published. Yi-Ming Weng and Kuan-Fu Chen performed the statistical analysis. Shou-Yen Chen helped to draft the manuscript. All authors read and approved the final manuscript.

## Pre-publication history

The pre-publication history for this paper can be accessed here:

http://www.biomedcentral.com/1472-6963/12/262/prepub
